# Efficacy and Safety in Retinal Vein Occlusion Treated with at Least Three Consecutive Intravitreal Dexamethasone Implants 

**DOI:** 10.1155/2016/6016491

**Published:** 2016-02-28

**Authors:** Julia Proença Pina, Khalil Turki, Julien Labreuche, Alain Duhamel, Thi Ha Chau Tran

**Affiliations:** ^1^Department of Ophthalmology, Claude Huriez Hospital, Michel Polonovski Street, 59037 Lille Cedex, France; ^2^Department of Ophthalmology, Orleans La Source Hospital, 14 rue de l'Hôpital, 45100 Orleans, France; ^3^Department of Biostatistics, EA2694, CHRU Lille, University of Lille, 59000 Lille, France; ^4^Department of Ophthalmology, Saint-Vincent de Paul Hospital, Lille Catholic University, Boulevard de Belfort, BP 387, 59020 Lille Cedex, France

## Abstract

*Purpose*. To evaluate the effects of repeated intravitreal dexamethasone implant (DI) (Ozurdex®) in eyes with macular edema (ME) due to retinal vein occlusion (RVO).* Methods*. Multicenter observational study including patients who received more than three consecutive DI on an “as-needed” basis for the treatment of ME in RVO.* Results*. A total of 18 eyes were included for analysis. Mean interval of retreatment with DI was 5.1 months between the first and second DI and 5.4 months following the second DI. Baseline BCVA was 0.74 ± 0.08 log-Mar; it significantly improved to 0.45 ± 0.04 2 months after the 3rd DI. There was no significant difference between the 3 first postinjection BCVA. CMT decreased from 617 *μ*m ± 120 *μ*m (baseline) to 330 ± 109 *μ*m two months after the third DI. Elevated intraocular pressure occurred in 50% and was controlled medically. Cataract progression leading to cataract surgery occurred in 69% of phakic eyes after a mean interval of 17 months.* Conclusion*. Repeated DI on an “as-needed” basis, with a retreatment interval <6 months, are effective in the long term in the management of ME due to RVO. Rates of increased intraocular pressure and cataract surgery seem to be higher than previously described when eyes were followed during a longer period.

## 1. Introduction

Retinal Vein Occlusion is the second most common retinal vascular disease after diabetic retinopathy. Macular edema due to branch retinal vein occlusion (BRVO) or central retinal vein occlusion (CRVO) is the main cause of visual loss in patients suffering from Retinal Vein Occlusion (RVO) [1, 2]. The pathogenesis of macular edema secondary to RVO is not yet well known.

Nonetheless some authors have underlined the key role of inflammatory cytokines and vascular permeability factors such as interleukin-6, prostaglandins, and VEGF [3, 4]. These factors are responsible for the breakdown of the blood-retinal barrier by dysregulation of endothelial cells. These recent advances in our understanding of the pathogenesis of ME have led to new therapies, including anti-VEGF agents and corticosteroids.

Dexamethasone posterior Segment Drug Delivery System: DEX PS DDS: Ozurdex (Allergan, Inc., Irvine, CA, USA) is a slow release, intravitreal, biodegradable dexamethasone implant that is injected through the pars plana by a customized, single-used applicator. The active drug, dexamethasone, is a corticosteroid with anti-inflammatory and anti-VEGF effects, and its intravitreal administration bypasses the blood-retinal barrier, allowing high intraocular concentration with minimal systemic absorption. The implant consists of a biodegradable copolymer matrix of lactic acid and glycolic acid, which enables the slow release of dexamethasone. It has been demonstrated that high concentrations of dexamethasone are sustained in the retina and vitreous during the first 2 months after the injection, and lower concentrations are sustained up to 6 months [5]. Intravitreal dexamethasone implant has been proven effective, approved by the regulatory agencies in the United States and Europe, and is currently used in clinical practice for the treatment of ME associated with RVO and noninfectious posterior uveitis [6]. It has also been demonstrated to be effective for the treatment of diabetic ME [7], Irvine-Gass syndrome [8], and ME secondary to retinitis pigmentosa [9]. The worldwide approval of Ozurdex in RVO followed results of an international 6-month study which investigated the effect of a single 0.35 or 0.7 mg Ozurdex injection compared with sham injection for treatment of macular edema in eyes with BRVO or CRVO (Geneva study) [10].

Most published studies of intravitreal dexamethasone implant (DI) focused on its short-term efficacy and safety, following patients for 6 or 12 months only [10–14]. Information regarding the response to multiple treatments, the optimal retreatment interval, and long-term follow-up is lacking. The purpose of this study is to evaluate the efficacy and safety of three or more intravitreal consecutive dexamethasone implant injections for the treatment of ME in RVO and administered on an “as needed” basis.

## 2. Material and Method

### 2.1. Patient Selection

We reviewed the charts of patients with decreased visual acuity due to RVO-related ME, who received at least three intravitreal Ozurdex injections in four retina clinics in France, between June 2009 and January 2014.

Inclusion criteria were (1) age > 18 years, (2) ME associated with CRVO or BRVO, (3) best corrected visual acuity (BCVA) between 20/400 and 20/32 (Snellen equivalent) at baseline examination, (4) central macular thickness (CMT) > 300 *μ*m, as measured by spectral-domain optical coherence tomography (SD-OCT) at baseline examination, and (5) need for more than 3 consecutive DI during the study period.

We excluded patients who underwent less than 3 DI during the study period and who had undergone previous surgery in the study eye in the last 6 months. Patients with additional ophthalmic comorbidity, which could have had a considerable influence on VA, were excluded from this analysis (advanced age-related macular degeneration, diabetic macular edema, proliferative diabetic retinopathy, and advanced glaucoma).

Informed consent was obtained in agreement with the Declaration of Helsinki for research involving human subjects in all institutions.

At baseline, all patients underwent a complete ophthalmic evaluation, including BCVA, tonometry, and SD-OCT (Spectralis, Heidelberg Engineering, Heidelberg, Germany) with CMT measurement.

Follow-up ophthalmic evaluations, including BCVA, fundus biomicroscopy, tonometry, and SD-OCT, were performed at the second month after each injection and bimonthly thereafter. Decision of retreatment was made in the discretion of the treating physician, based on decreased visual acuity and/or recurrence of ME, which was documented by SD-OCT (intraretinal and/or subretinal fluid, CMT > 300 *μ*m). He also decided the need to continue with DI and whether to add other medical and/or laser treatments in case of incomplete responsiveness and/or ME recurrence.

Demographic data of the pooled patients, duration of RVO, and previous treatments were collected. Outcome measures included mean change in BCVA and CMT from baseline to two months after first, second, and third DI and at the time of ME recurrence. The proportion of injections with at least 3 lines of BCVA improvement and the proportion of injections exhibiting ≥3 lines of BCVA worsening, retreatment interval between the 1st and 2nd DI, between the 2nd and the 3rd DI, and the incidence of side effects following repeated DI were recorded.

### 2.2. Statistical Analysis

Visual acuity values were converted to the LogMar scale for statistical purpose. According to Holladay and the University of Freiburg study group results, blindness was set at 0.00125/2.9 (decimal/LogMar), light perception was set at 0.0025/2.6, hand movements were set at 0.005/2.3, and counting fingers were set at 0.014/1.85 [15, 16].

We assessed the effect of injection of dexamethasone on visual acuity and central macular thickness values using an analysis of variance for repeated measures. We used a linear mixed model in order to take into account the correlations between the repeated measures and the existence of missing data. Statistical testing was done at the two-tailed *α* level of 0.05. Data were analyzed using the SAS software package, release 9.3 (SAS Institute, Cary, NC).

## 3. Results

Results were summarized in Tables 1 and 2. During the study period, 18 eyes of 17 patients (median age, 70 years (range, 36–94); 8 males, 9 females) met the inclusion and exclusion criteria. All patients are Caucasian. Data were collected retrospectively in four hospitals caring for retinal diseases. Of the 18 eyes, 11 (61%) had ME secondary to CRVO and 7 (39%) had ME secondary to BRVO. The median duration of ME was 2.8 months (range, 0.5–10) before treatment. Dexamethasone implant was first-line treatment in 11 cases (65%). Among the 7 eyes treated by other therapies previously, 4 eyes were treated with anti-VEGF injections (bevacizumab), 1 eye was treated with both anti-VEGF and triamcinolone, and 2 eyes were treated with triamcinolone. Ten eyes (55%) received either macular grid laser treatment (*n* = 5) or retinal panphotocoagulation (*n* = 5). Ten patients (58%) had history of high blood pressure. One eye was initially treated for glaucoma by monotherapy at the beginning of the disease. Mean follow-up time was 17 ± 4.9 months (period of DI treatment only).

### 3.1. Visual Acuity and Repeated Dexamethasone Injections

**Figure 1:**
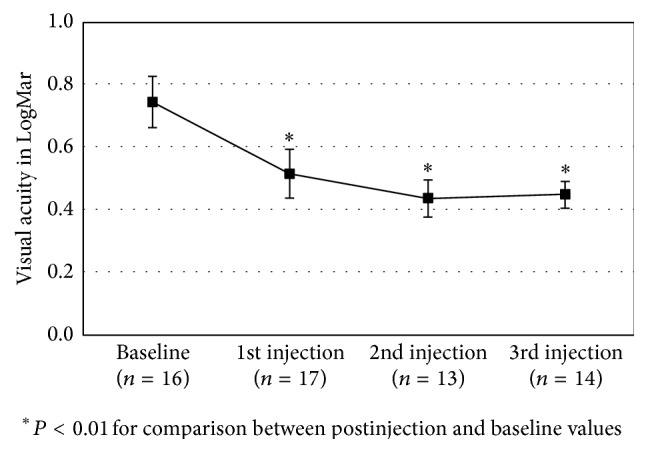
Mean (±SE) LogMar visual acuity at baseline and after 2 months of each injection of dexamethasone.

As shown in Figure 1, each injection was associated with a gain in visual acuity (*P* < 0.01 for all comparisons with baseline value). There was no significant difference between the 3 postinjection visual acuity measures (*P* = 0.63). The mean (±SE) LogMar BCVA at baseline was 0.74 ± 0.08 and increased to 0.45 ± 0.04 2 months after the 3rd DI, yielding an overall mean increase of 0.30 (95% CI, −0.50 to −0.08, *P* = 0.009). The mean difference in LogMar values after 3 consecutive injections was not significantly different between CRVO and BRVO cases (−0.35 versus −0.23, *P* = 0.56). No difference was found between treatment-naive and no-treatment naïve eyes (Table 1). Eight of 18 eyes (44%) showed ≥3 lines of improvement after repeated intravitreal DI, while 1 of 18 (5%) eyes exhibited ≥3 lines of worsening from baseline BCVA. An improvement of 15 letters or more after DI was achieved for 38% of the 65 injections done. A loss of 15 letters or more was observed in 2% of injections.

### 3.2. Central Macular Thickness and Repeated Dexamethasone Injections

**Figure 2:**
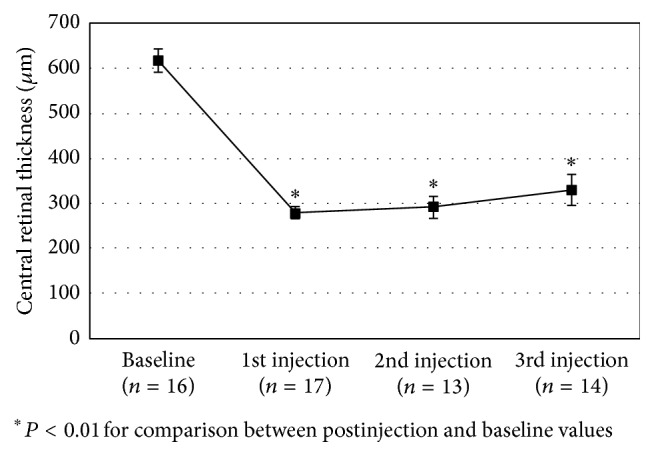
Mean (±SE) central retinal thickness at baseline and after 2 months of each injection of dexamethasone.

As shown in Figure 2, each postinjection CMT value was significantly lower than baseline values (all *P* < 0.001). Difference between the 3 postinjection CMT values did not reach the significance level (*P* = 0.082), a trend toward a lower effect with number of injections found; the mean difference (±SE) from baseline was −337 ± 29 *μ*m after 1st injection, −19 ± 31 *μ*m after 2nd injection, and −282 ± 30 *μ*m after 3rd injections.

The overall mean decrease in CMT values after the 3 consecutive injections was 282 *μ*m (95% CI, 220 to 343, *P* < 0.001). The mean change in CMT observed after 3 injections was not different between CRVO and BRVO cases and between treatment-naive and no-treatment naïve eyes (Table 2). In each subgroup, a significant change from baseline to 3rd injection was found.

### 3.3. Timing of Reinjection

The mean time of reinjection between first and second one was 5 ± 1.3 months and between the second and third DI was 5.4 ± 1.7 months (range, 3–9). Eight eyes of the cohort were injected more than 3 times consecutively (4 to 6 times). Interval between the third and fourth injection was 5.3 ± 1.3 months.

### 3.4. Safety of Repeated Injections

One eye was treated for glaucoma before DI with a good control of intraocular pressure by monotherapy. During the study period, IOP increases occurred in 9 eyes (50%) and were well controlled medically by mono- or bitherapy. Cataract progression leading to cataract surgery occurred in 69% of phakic eyes (9 of 13 eyes). Mean interval between the first DI injection and cataract surgery was 17 ± 9 months (ranged from 5 to 34 months). No cases of retinal detachment, vitreous hemorrhage, or endophthalmitis were encountered in any of these patients.

## 4. Discussion

We aim to evaluate the long-term outcome of ≥3 consecutive DI (range from 3 to 6) in ME-related RVO from routine clinical practice, on an “as needed basis.” The results of our study are summarized as follows: (1) each injection leads to stabilization of visual acuity (98%) and reduction of CMT in the overall RVO population; (2) repeated DI is effective in previously treated eyes with either anti-VEGF therapy or triamcinolone or grid laser; (3) repeated DI is effective in both CRVO and BRVO; (4) interval retreatment is less than 24 weeks with an interval of 5 ± 1.3 months between the first and the second DI and 5.4 ± 1.7 months between the second and the third DI; (5) side effects rate is higher in this study since patients received more injections and had a longer follow-up duration.

Our results show that DI is still effective after third injection. A marked improvement of 15 letters or more was recorded after the first 3 DI in 38% of procedures. A loss of 15 letters or more was observed only in 2% injections. Functional results were similar to those of previous studies [10, 17–19]. CMT was significantly reduced by 282 *μ*m (95% CI, 220 to 343, *P* < 0.001) which represents a reduction of 45.7% (*P* < 0.001) after the first 3 consecutive injections, as previously reported [12, 17, 19]. Visual gain and mean changes in CMT observed after 3 injections were not different between CRVO and BRVO eyes. Functional and morphological responses were still observed in repeated DI and there was no tachyphylaxis phenomenon. In retrospective single center of 33 eyes receiving at least 2 DI, Querques et al. found that repeated treatment is effective with improvement of visual acuity and reduction of CMT, and the peaking efficacy was found at 1.8 months from the 2nd DI [19]. In a multicenter retrospective study including 128 eyes, Coscas et al. [17] found also visual gain and reduction of CMT after the second injection. Whereas the last author found that repeated DI injections achieved a better mean visual gain in treatment-naïve eyes than previously treated eyes (visual gain 0.26 ± 0.36 versus 0.04 ± 0.26, *P* = 0.03), this finding was not observed in our study or in Querques et al.'s report [19]. There was no difference in CMT reduction after repeated DI injections between treatment-naïve eyes and previously treated eyes in our study, as was in previous report [17, 19].

Meanwhile, in the Geneva study, retreatment was recommended 6 months after the first injection. However, recurrence is high (91%) before 6 months in most reports whose intervals range from 3.2 to 8.7 months [13, 17, 19–22]. Early retreatment after 16 weeks instead of 24 weeks was indicated in 50% to stabilize the improved functional and anatomical results [12]. Time of reinjection between each retreatment is less than 6 months in our study.

In our study, median duration of ME was 2.8 months (range, 0.5–10) before treatment. Several studies and post hoc analysis of Geneva trials have underlined that duration of ME is an independent predictor of the response to treatment with DI in patients with vision loss resulting from ME of at least 6 weeks of duration resulting from RVO [10, 23]. Delaying treatment by even 1 month is associated with a significant decrease in the likelihood of achieving a clinically relevant improvement in BCVA (at least a 15-letter gain) or reduction in central retinal thickness (by at least 200 *μ*m) in this patient population [23]. Other independent predictors were older age, better BCVA at time of treatment, and presence of CRVO rather than BRVO. However, only duration of ME is a modifiable risk factor that can be taken into account when deciding on a course of treatment. This data piece is now rather well-known by treating physicians who, most of the time, respected a period of observation below 3 months.

In the Geneva study, cataract progression was found in 29.8% of patients after 1 year with 2 DI, cataract extraction was needed in only 1.3% of eyes, and IOP > 25 mmHg was found in 16% of patients after one DI [18]. The safety profile after ≥3 DI injections was different with higher adverse events rate than previously reported since cataract surgery was needed in 69% of phakic eyes and IOP increased in 50% of eyes [10, 11, 17–19]. However, in previous studies, cataract progression was evaluated after one or two DI and the observation was interrupted 6 months after the last injection. Indeed, in E. Moisseiev's study including 17 patients who received 1 to 2 DI, 10/17 (58.8%) had cataract progression and 35.2% had undergone phacoemulsification after a mean follow-up of 50.5 months [24]. These results were consistent with ours: DI leads to cataract surgery more frequently than firstly reported after more than one year of follow-up. In fact, cataracts are a known complication of all types of steroid administration routes and occur more commonly in patients with long-term steroid use [25]. In our report, the higher rate of cataract surgery might be explained by a long period of follow-up, and surgery was performed after a mean interval of 17 months from the first DI injection. Cataract extraction is a safe and simple surgery and should be considered as part of the treatment process of a long-term disease. The rate of IOP increases was also higher (50%) in our study than previously published [10, 17–19]. However, the IOP elevations were mild, transient, and well controlled by medication; no patient needed filtering surgery. This high rate of IOP increases was found in some reports. In a one-year prospective study including 16 patients treated with DI and followed during 12-month period, 50% of patients had an increase ≥10 mmHg [26]. In the Shasta study which is a multicenter chart review, 32.6% of 289 eyes receiving at least 2 DI developed intraocular increase (≥10 mmHg), 29.1% used medication, and 1.7% required surgery [27]. Secondary glaucoma after intravitreal injection of DI might be underestimated in the Geneva studies. This finding is important since secondary glaucoma induced by DI is the main reason for leading the decision to therapeutic change. These also explained difficulties we met to find charts which fulfilled the inclusion criteria of the study.

Although limited by the retrospective nature and the small numbers of patients included, our study shows “real-life” results of ≥3 DI retreatments in ME-RVO related and provides useful comparisons with the results of previous study.

In conclusion, our results confirmed that good functional and anatomic response remains after ≥3DI, on an “as needed basis,” with a retreatment interval <6 months. However, the clinical safety profile reported here calls for frequent IOP control if DI is applied as a long-term treatment.

## Figures and Tables

**Table 1:** Mean (±SE) LogMar visual acuity before and after 3 consecutive injections of dexamethasone, overall and according to the occlusion type and previous therapy.

	Baseline^*∗*^	2 months after 3rd injection	Mean change (95% CI)	*P* ^†^	*P* ^‡^
Overall (*n* = 18)	0.74 (0.08)	0.45 (0.04)	−0.30 (−0.50 to −0.08)	0.009	
Occlusion type					
CRVO (*n* = 11)	0.81 (0.14)	0.46 (0.05)	−0.35 (−0.70 to −0.002)	0.049	0.56
BRVO (*n* = 7)	0.66 (0.04)	0.43 (0.10)	−0.23 (−0.46 to −0.004)	0.047	
Treatment-naïve eyes					
No (*n* = 11)	0.71 (0.10)	0.44 (0.06)	−0.28 (−0.56 to 0.004)	0.053	0.80
Yes (*n* = 7)	0.80 (0.15)	0.47 (0.08)	−0.39 (−0.77 to −0.001)	0.049	

CRVO: central retinal vein occlusion branch; BRVO: branch retinal vein occlusion branch.

Linear mixed regression analysis was used to estimate the mean change and to perform the between comparison.

^*∗*^Defined as first LogMar values before 1st injection of dexamethasone.

^†^ *P* for comparison between baseline and postinjection values.

^‡^ *P* for between-group comparison calculated by including interaction term between groups and time of measure in linear mixed model.

**Table 2:** Mean (±SE) central retinal thickness before and after 3 consecutive injections of dexamethasone according to the occlusion type and previous therapy.

	Baseline^*∗*^	2 months after 3rd injection	Mean change (95% CI)	*P* ^†^	*P* ^‡^
Overall	617 (26)	330 (34)	−282 (−342 to −220)	<0.001	
Occlusion type					
* *CRVO (*n* = 11)	648 (31)	325 (45)	−317 (−413 to −222)	<0.001	0.27
* *BRVO (*n* = 7)	568 (44)	339 (58)	−229 (−428 to −30)	0.033	
Treatment-naïve eyes					
* *No (*n* = 11)	642 (31)	382 (52)	−254 (−370 to −138)	0.001	0.41
* *Yes (*n* = 7)	577 (46)	261 (19)	−316 (−447 to −186)	0.001	

CRVO: central retinal vein occlusion branch; BRVO: branch retinal vein occlusion branch.

Linear mixed regression analysis was used to estimate the mean change and to perform the between comparison.

^*∗*^Defined as first central retinal thickness values before 1st injection of dexamethasone.

^†^ *P* for comparison between baseline and postinjection values.

^‡^ *P* for between-group comparison calculated by including interaction term between groups and time of measure in linear mixed model.
